# MR‐linac is the best modality for lung SBRT

**DOI:** 10.1002/acm2.12615

**Published:** 2019-05-21

**Authors:** Andrew Godley, Dandan Zheng, Yi Rong

**Affiliations:** ^1^ Miami Cancer Institute Baptist Health South Florida Miami FL USA; ^2^ Department of Radiation Oncology University of Nebraska Medical Center Omaha NE USA; ^3^ Department of Radiation Oncology University of California Davis Comprehensive Cancer Center Sacramento CA USA

## 1 INTRODUCTION

The introduction of MR‐linac hybrid modality started a new era in image guidance for radiotherapy. It provides superior soft tissue contrast compared to conventional x‐ray imaging, and offers the ability to track and gate treatment delivery, yet challenges and limitations remain in various aspects.^1^ Our previous Parallel Opposed issues discussed the additional physicist qualification and residency requirements for the use of MRI in radiotherapy settings.^2^, ^3^ Herein, we continued our discussion on the clinical use of MR‐linac and its potential improvement in treatment efficacy for lung stereotactic body radiotherapy (SBRT). Dr. Andrew Godley believes that “MR‐linac is the best modality for lung SBRT,” while Dr. Dandan Zheng explained her doubts and concerns.

Dr. Andrew Godley received his Ph.D. in High Energy Physics from the University of Sydney in 2001. Dr Godley continued in particle physics at the University of South Carolina and Fermilab, until 2007 when he trained in medical physics at the Medical College of Wisconsin. Dr. Godley spent 7 years as a staff physicist at the Cleveland Clinic before joining the Miami Cancer Institute in 2018 to help develop their MR‐linac and SBRT programs.

Dr. Dandan Zheng received her Ph.D. in Applied Science from the University of California Davis in 2007. After conducting a postdoctoral training in Radiation Oncology at Virginia Commonwealth University, she joined the department as an assistant professor in 2009. In 2012 Dr. Zheng moved to University of Nebraska Medical Center where she is currently an associate professor and the medical physics residency program director.  Dr. Zheng is an avid peer reviewer/associate editor for many journals, and her research interests include image guidance, radiomics and machine learning, stereotactic RT, dose algorithms, and motion management.

## 2 OPENING STATEMENT

### 2.1 Dr. Andrew Godley

One vision of future radiotherapy is for patients to arrive for treatment, lie down on the couch, and the treatment system to automatically locate and irradiate their tumor, regardless of its position or motion, sparing all but the surrounding few millimeters of normal tissue. The hybrid technology of MR‐guided linear accelerator is today's embodiment of that vision. It provides three main advantages to patients: monitoring the actual tumor location during treatment delivery, comfort, and accuracy. These advantages would benefit any patient undergoing radiotherapy, but provide specific gains for lung SBRT patients. Clinical trials have shown lung SBRT already achieves 98% tumor control, however there is still a 17% risk of grade 3–4 toxicity^4^ and it requires oppressive patient immobilization. The fast‐growing number of patients eligible for lung SBRT further presses the need for technological improvement. The MR‐linac tracking and accuracy reduce the volume of normal lung and chest wall irradiated, decreasing toxicity, and obviating the need for immobilization.

MR‐linac provides the best targeting of any system available as it directly images the tumor throughout treatment delivery. The reasons are four‐fold: First, 2D cine‐MRI based tumor tracking enables gating directly on the tumor position relative to the planned position. This cine imaging incurs no additional radiation exposure. Although some conventional systems acquire planar imaging during treatment, the radiation dose over a large area would be excessive to achieve the continuous monitoring of the MR‐linac. Second, while using external surrogates facilitates continuous monitoring, these surrogates require at least an additional 1 mm margin to ensure correlation with the tumor.^5^ Third, the precise gating window of the MR‐linac provided by its continuous tumor tracking limits or eradicates multileaf collimator (MLC) interplay issues, which was reported to affect delivery accuracy of other modalities^6^ and thus reduce target coverage.^7^ Lastly, the tumor based gating eliminates the need to define an internal target volume (ITV), which is conventionally used to ensure tumor coverage during the breathing cycle, but increases the volume of normal lung irradiated. Further, traditional ITVs are just a snapshot of tumor motion observed over tens of seconds in a 4D CT. When compared to a treatment length cine‐MRI based ITV, the four‐dimensional computed tomography (4D CT) based ITV typically captures only 45%–90% of the required volume,^8^ leading to potential under dosing. The MR‐linac continuous tracking accounts for the breathing motion including large excursions of the tumor, so that this motion does not have to be forcibly limited.

With the above‐mentioned target monitoring and tracking, immobilization for MR‐linacs does not then feature compression or breath control, leading to higher patient comfort during treatment. Due to the continuous imaging of the tumor, the patient can be coached by the therapists during treatment, or even see and respond to their own cine‐MRI, in order to achieve optimal breath holds. By consistently placing the tumor in the gating window, the gating duty cycle is increased and treatment times reduced. Video feedback for breath‐hold delivery has been positively viewed by patients treated on the MR‐linac.^9^ Commonly no immobilization is necessary, with the patient arranged comfortably on the couch. No implanted fiducials are required either, making for a safer and noninvasive treatment. This increased patient comfort is achieved without limiting the accuracy of the MR‐linac.

The MR‐linac allows several other major improvements in terms of accuracy in planning and treatment. The treatment planning algorithm used for MR‐linac planning has always been based on Monte Carlo simulation, which provides a more accurate dose estimation than used by many conventional SBRT planning systems.^10^ In addition, the pretreatment volumetric MR imaging can be acquired in 17 s, not only does this make for an easier breath hold than for current cone‐beam CTs, but it also reduces averaging of the tumor due to motion from repeated or longer breath holds. This leads to more accurate alignment of the patient in the MR‐linac. Furthermore, the minimum 4 Hz rate of cine‐MRI allows for the capture of cardiac derived tumor motion for central lesions, something that would be missed by all other means of intrafractional lung imaging, and would lead to higher toxicities or under coverage of the tumor. This fast rate of tumor imaging can enable MLC tracking,^11^ which currently relies on implanted fiducials and portal images that are often blocked by the MLC. Finally, an MR‐linac can provide diagnostic sequences daily, such as dynamic contrast enhanced and diffusion weighted, to observe treatment response.^12^, ^13^ Observed changes in tumor volume coupled with tumor response data can inform dose adaption, to provide the optimal curative dose.

As MR‐linacs become more prevalent, these advantages will be borne out in improved outcomes for our lung SBRT patients. Any disadvantages of MR‐linacs will be eroded as the technology advances. While conventional linacs have achieved excellent lung tumor control rates, their lack of tumor imaging during treatment has left us irradiating unnecessarily large volumes of normal lung tissue and chest wall. Thankfully we now have the technology to alleviate this and continue the success of lung SBRT with the MR‐linac.

### 2.2 Dr. Dandan Zheng

Since its clinical introduction, the MR‐linac has quite often attracted the spotlight as the state‐of‐the‐art treatment modality in the era of image‐guided adaptive radiotherapy. MRI‐guidance may provide the much needed breakthrough for disease sites such as pancreas, where other radiotherapy treatment modalities lack efficacy due to dose‐limiting normal tissue toxicity, poor on‐board x‐ray contrast, and weak internal motion‐external surrogate correlation, etc. When it comes to the titled statement for lung SBRT, however, I cannot agree that the MR‐linac represents the best modality for reasons elaborated below.

#### 2.2.1 Technical limitations of the linac component

Integrating MRI and linac poses tremendous technical challenges on both systems.^14^, ^15^, ^16^, ^17^, ^18^ While the integration has finally been made feasible after decades of R&Ds, the linac component in an MR‐linac is still not up to the highest standard of modern linacs. The essential technologies pertaining to treatment time reduction for lung SBRT, such as volumetric modulated arc therapy (VMAT) and high dose rates (up to 2400 MU/min),^19^, ^20^ are not available on the MR‐linac. Currently, the MR‐linac is only capable of step‐and‐shoot, low dose rate (450 MU/min for Elekta Unity or 600 MU/min for ViewRay MRIdian), low gantry speed, and low MLC speed, all of which substantially lengthen the treatment duration. This prolonged treatment time poses contraindication for patients who cannot tolerate due to back pain or poor performance status, and increases treatment variations and motion uncertainty. In addition, the MR‐linac has a limited lateral range of isocenter and forces off‐axis geometry for those tumors located outside the range. This can potentially lead to excessive monitor units and hence high integral dose to normal tissue.^21^ The couch correction range is also limited with no option of couch rotation and, in some models, lateral translation. Furthermore, the electron return effect is much more pronounced in lung and can lead to considerable dose distortion at tissue‐air interfaces. Some early studies have shown increased dose in lung and skin, as well as compromises to the accuracy of both patient plan and QA dosimetry.^11^, ^22^, ^23^

#### 2.2.2 Technical limitations of the MR component

The most desirable features to those early adopters might be the real‐time internal imaging capability with an MR scanner. However, cautions should be taken as they are not standard diagnostic MR scanners either. First, the MR‐linac often uses lower magnetic field strengths than diagnostic MRIs (e.g., ViewRay MRIdian uses a 0.35 T scanner), therefore rendering compromised MR image quality. Second, the limited real‐time tracking range excludes those cases with very peripheral lesions. Third, real‐time target monitoring is based on 2D MR cine images, therefore omitting the volumetric or the third‐dimension motion information. Fourth, the MR sequences typically used for lung are prone to irregular breathing artifacts and banding artifacts.^24^ Fifth, in the desirable adaptive radiotherapy application, MRI cannot provide the CT‐number information for heterogeneity correction which is especially important for lung SBRT dose calculation. Any method to address this fundamental limitation introduces additional uncertainties which are more pronounced for lung because lung and bone feature little MR signal.^25^ Last but not least, tissue heterogeneity in lung can induce pronounced geometric distortions in MR images due to local susceptibility differences, and these errors are in addition to other MR distortions such as those due to main magnetic field and gradient field nonlinearity.^26^

#### 2.2.3 Incompatible patient populations

For the patient population with any contraindication against MRI, the MR‐linac is not an option. These include patients with claustrophobia and those with ferromagnetic implants such as prostheses, cardiac pacemakers and defibrillators, artificial heart valves, and cochlea implants. In addition, due to the bore size limitation, the MR‐linac also excludes large patients and prevents the use of many immobilization devices and body positions.

#### 2.2.4 Alternative modalities

Currently, there are many successful alternative modalities and technologies for lung SBRT that the MR‐linac cannot rival. As mentioned previously, standard linacs are capable of delivering highly conformal lung SBRT plans at very high speeds with VMAT and high dose rates.^19^ Less uncertainty and higher accuracy are further ensured with the combinations of various imaging options (such as cone beam CT (CBCT), 4D CBCT, fluoroscopy, and triggered MV/kV imaging) as well as gating or tracking mechanisms (such as external fiducial‐ or surface‐based optical tracking and internal fiducial‐based radiofrequency tracking). Tumor motion can also be mitigated by breath‐holding techniques such as active breathing control, compression techniques, and audio‐visual coaching with feedback. In addition, target dosimetry can be optimized with noncoplanar geometries and 6‐degree‐of‐freedom couch corrections. Furthermore, systems such as CyberKnife^TM^ have demonstrated extensive success using real time tumor tracking with combined internal and external tracking.^27^

#### 2.2.5 Health economics

MR‐linacs are expensive at a price point 2–4 times of standard linacs and therefore remain questionable as a cost‐effective modality for value‐based health care. Its cost could be more justified in diseases such as pancreatic cancer where the current treatment efficacy is poor and x‐ray based imaging does not provide sufficient contrast. Whereas the excess cost may not be justified for lung SBRT where the standard treatment modalities are much more cost‐effective and highly successful with >90% local control rates and low toxicity to normal tissues, along with adequate image‐guidance and tracking.^27^, ^28^

In conclusion, MR‐linac is clearly not a viable lung SBRT treatment modality for a large population of patients with contraindications. For the other patients, it is still not the best modality based on the arguments above, as the alternative modalities provide more advanced dosimetry and delivery efficiency, more robust and versatile image guidance and motion management approaches, clear cost‐effectiveness, and well‐documented clinical success.

## 3 REBUTTAL

### 3.1 Dr. Andrew Godley

I would like to thank my colleague and friend Dr. Zheng for eloquently sharing her concerns with MR‐Linac lung SBRT. I am happy to now assuage those concerns. The first of which is treatment time. While treatment *delivery* is the one advantage of conventional linacs, due to the extra time required for patient setup, immobilization, image guided radiotherapy (IGRT) (especially if multiple imaging modalities are used), and gating a conventional linac SBRT lung treatment still ends up in the same 45 min timeslot as our MR‐linac SBRT. Technical limitations are not solely the bugbear of MR‐linacs. Conventional linacs have limited gantry and couch angles for lateral tumors, and Cyberknife is limited in utility for posterior tumors. While there are increased MU for off‐axis MR‐linac treatments, the double stack MLC means there is no increase in leakage radiation. The article cited^21^ in fact finds no other issues with off‐axis treatment. In terms of aligning the patient after imaging, commercial MR‐linacs either shift the couch or shift the MLC and rotational corrections are not dosimetrically important for lung SBRT.^29^ Electron return effect is accounted for in the Monte Carlo dose planning and there is only an increase for skin in standard fractionation, not SBRT.^22^ The article however confirms lung dose is lowered due to reduced margins of lung SBRT on the MR‐linac.

Perplexingly, Dr. Zheng suggests imaging as a weakness of MR‐linac by comparing it to diagnostic MR, rather than the low quality CBCT or planar kV conventional linacs rely on. MR‐linacs achieve spatial accuracy of 1–2 mm in a 35 cm diameter sphere.^30^ Their 70 cm bore allows for lateral patient shifts in order to ensure all targets can be within this spatially accurate region and will track well. Breath holds are used to ensure the 3D MR image is not affected by motion. While adaptive lung SBRT is not as common as other sites, it can certainly be accomplished on the MR‐linac^31^ and CBCTs also lack correct electron density. MRI has been in use since the 1970s, as such the majority of modern medical implants have been manufactured to be MR safe including all joint implants and recent implantable cardiac devices. With the low field strength of MR‐linacs, many more are compatible. Patients undergoing any radiotherapy can have anxiety, and are medicated as needed.

My opening statement addressed the advantages of the MR‐linac over current modalities. In particular, it mentioned the extra radiation required for CBCT (4D!) and fluoroscopy. The only modality Dr. Zheng discussed that could provide continuous tumor monitoring is radiofrequency tracking, but this requires an invasive procedure to implant the markers. Cyberknife is limited in size and position of tumors for its marker‐less tracking.^32^ All the other techniques are only surrogates for the tumor position. Lastly Dr. Zheng mentions cost. To the patient there is no difference in cost. The MR‐linac purchase cost is actually only 1–2 times a conventional linac, depending on how many extras like 4D CBCT, radiofrequency tracking, 6DoF couch, or gating you buy in an attempt to reach the tracking ability of the MR‐linac.

In summary, the only compelling advantage of conventional linacs is their delivery speed, but without continuous tumor tracking, this just allows you to miss the target quicker. Hence I maintain that MR‐linac is the best modality for lung SBRT.

### 3.2 Dr. Dandan Zheng

I, too, share the vision of Dr. Godley's on the ideal radiotherapy treatment. But today's MR‐linac is far from the embodiment of that vision. More importantly, while the MR‐linac should be further advanced as a frontrunner modality toward that vision for diseases like pancreatic cancer, it is not the case for lung SBRT. For pancreas, current radiotherapy provides relatively poor efficacy with organ toxicity partly due to low soft‐tissue imaging contrast, while MRI may offer the important soft‐tissue contrast resolution that is superior to the competing x‐ray technologies. Whereas for lung, contrast is not as challenging, and current SBRT can already achieve 98% tumor control as cited by Dr. Godley.^4^ As discussed above and will be stressed again below, numerous existing technical issues in MR‐linacs make it far from being the ultimate solution toward that vision. But even hypothetically assuming all issues would have been perfectly resolved someday, arguing for the MR‐linac as the best modality for lung SBRT would still be like arguing for proton therapy for prostate: Are there enough clinically meaningful gains to justify the higher cost?

Let's revisit MR‐linacs' three main advantages laid out by Dr. Godley.

#### 3.2.1 Comfort

With VMAT, high dose rates and fast collimators, lung SBRT treatment delivery time can be dramatically shortened to 2–3 min on standard linacs. Due to the magnetic field, MR‐linacs pose technical limitations that lengthen the treatment by several folds. On top of that, MR‐gating can prolong treatment delivery even more. For a treatment that currently takes minutes on standard linacs and may someday take seconds with FLASH‐RT,^33^ how would its hour‐long gated counterpart on MR‐linacs increase patient comfort?

#### 3.2.2 Accuracy

In the opening statement Dr. Godley described, “MR‐linac provides the best targeting of any system available as it directly images the tumor throughout treatment delivery.” This statement is misleading because it assumes a huge leap of faith. Being able to “image” the tumor real time is far from being able to “target” it real time. TG76 summarizes respiratory motion management in four main approaches: (a) motion‐encompassing, that is, ITV; (b) respiratory gating, (c) voluntary or involuntary breath hold (including compression); and (d) real‐time tumor‐tracking.^34^ Dr. Godley described MR‐tracking as real‐time tumor‐tracking, the most desirable approach from the above list, where it “continuously accounts for the breathing motion including large excursions of the tumor.” But this mode is currently unavailable on MR‐linacs as only the first of four necessary conditions is provided for real‐time tumor‐tracking that is, identifying the tumor position in real time. MR‐linacs offer no solution for the other three critical requirements: motion prediction to address latency, repositioning of the beam, and dose adaptation for changing anatomy. With the presence of the magnetic field, repositioning of the beam will be particularly challenging. In contrast, CyberKnife^TM^ has already developed a working real‐time tracking solution using its robotic linac and Synchrony^TM^ system and had years of documented clinical success.^27^ Meanwhile, standard linacs also have developed solutions such as MLC‐tracking and couch‐tracking,^35^, ^36^ but these solutions would be difficult to adopt on MR‐linacs due to the interfering magnetic field. The so‐called “MR‐tracking” is not really “tracking” but gating, which substantially lengthens the treatment duration and has fallen out of fashion for lung SBRT because of it. In fact, the MR‐gating may actually be managing the extra motion uncertainty created by the lengthened treatment but does not necessarily improve the overall accuracy. The much larger (compared with the conventional ITV) MR‐ITV created with treatment‐length cine MRIs that Dr. Godley described earlier might serve as a proof to this.^8^ Since the current high tumor control rate argues against the tumor underdosing using the conventional ITV that Dr. Godley cautioned about, I would offer another more likely reason for the larger MRI‐ITV volumes: an effect of the increased motion uncertainty due to the much longer “treatment length”!

#### 3.2.3 Tumor monitoring

Although incapable of real‐time tumor targeting, the expensive MR‐linac does provide the desirable real‐time tumor monitoring. However, for lungs, such monitoring capability is not unique to MRI due to high x‐ray imaging contrast and minimally invasive fiducial implantation procedures. Continuous x‐ray monitoring based on tumors or fiducials has long been established and achieved high precision for lung. Significantly shortened treatment delivery and SBRT hypo‐fractionation have further alleviated concerns about additional radiation exposure. Meanwhile, exposure‐free radiofrequency tracking has been FDA‐approved for lung and can be integrated with real‐time MLC‐tracking.^35^ In addition to these mature technologies, the feasibility of other real‐time lung‐tumor monitoring has also been demonstrated with radiation‐dose‐free Compton‐scatter imaging using the therapy beam^37^ and with PET emission guidance.^38^

In summary, MR‐linacs should be better used where they are needed. For lung SBRT, competing technologies are highly successful, and offer higher cost‐effectiveness and better solutions.

## CONFLICT OF INTEREST

No conflicts of interest.
